# Modification of distinct ion channels differentially modulates Ca^2+^ dynamics in primary cultured rat ventricular cardiomyocytes

**DOI:** 10.1038/srep40952

**Published:** 2017-01-19

**Authors:** Xichun Li, Liping Shen, Fang Zhao, Xiaohan Zou, Yuwei He, Fan Zhang, Chunlei Zhang, Boyang Yu, Zhengyu Cao

**Affiliations:** 1 State Key Laboratory of Natural Medicines & Jiangsu Provincial Key laboratory for TCM Evaluation and Translational Development, China Pharmaceutical University, Nanjing, Jiangsu, 211198 China

## Abstract

Primary cultured cardiomyocytes show spontaneous Ca^2+^ oscillations (SCOs) which not only govern contractile events, but undergo derangements that promote arrhythmogenesis through Ca^2+^ -dependent mechanism. We systematically examined influence on SCOs of an array of ion channel modifiers by recording intracellular Ca^2+^ dynamics in rat ventricular cardiomyocytes using Ca^2+^ specific fluorescence dye, Fluo-8/AM. Voltage-gated sodium channels (VGSCs) activation elongates SCO duration and reduces SCO frequency while inhibition of VGSCs decreases SCO frequency without affecting amplitude and duration. Inhibition of voltage-gated potassium channel increases SCO duration. Direct activation of L-type Ca^2+^ channels (LTCCs) induces SCO bursts while suppressing LTCCs decreases SCO amplitude and slightly increases SCO frequency. Activation of ryanodine receptors (RyRs) increases SCO duration and decreases both SCO amplitude and frequency while inhibiting RyRs decreases SCO frequency without affecting amplitude and duration. The potencies of these ion channel modifiers on SCO responses are generally consistent with their affinities in respective targets demonstrating that modification of distinct targets produces different SCO profiles. We further demonstrate that clinically-used drugs that produce Long-QT syndrome including cisapride, dofetilide, sotalol, and quinidine all induce SCO bursts while verapamil has no effect. Therefore, occurrence of SCO bursts may have a translational value to predict cardiotoxicants causing Long-QT syndrome.

The orchestrated mechanical activity of the heart is controlled by electrical pulses initiating from the sino-atrial node and finally conveys to the ventricles leading to rapid depolarization of all ventricular myocytes and coordinated contraction of the heart^1^. The rhythmic cardiac activity can be disrupted under certain circumstances, leading to cardiac arrhythmia. Both abnormally slow (bradycardia) and rapid (tachycardia) heart rates can lead to syncope and sudden death^1^,^2^. The most dangerous arrhythmias are those that originate from the ventricles, such as torsades de pointes (TdP) ventricular tachycardia and ventricular fibrillation^3^,^4^. Many studies have demonstrated that gain or loss of function of ion channels could shape cardiac action potentials (APs) and contribute to arrhythmia susceptibility^5^. Voltage-gated sodium channels (VGSCs) are responsible for the AP generation of the cardiomyocytes. Dysfunction of VGSCs by point mutation on the α-subunit leads to several types of arrhythmia, such as Long-QT (LQT) syndrome and Brugada syndromes^6^,^7^. Voltage-gated potassium channels (VGPCs) participate in the repolarization of the AP. Loss-of-function of Kv conductance results in AP prolongation leading to LQT syndrome while gain-of-function results in shortened AP duration leading to Short QT (SQT) syndrome^8^. Among the Kvs, the hERG channels (Kv11.1, encoded by human *Ether-à-go-go* Related Gene) are the major contributors to rapid delayed rectifier potassium currents (I_Kr_) which are involved in AP repolarization^9^. In many cases, inhibition of hERG channels results in prolonged AP leading to LQT ventricular arrhythmia, and sometimes, sudden cardiac death^10^. Therefore, functional alteration of the sodium and potassium channels tightly associated with the arrhythmia^11^.

Primary cultured cardiomyocytes show spontaneous transient increase in intracellular Ca^2+^ concentration (spontaneous Ca^2+^ oscillations, SCOs)^12^. These SCOs occur parallel with the AP generation and control ventricular cardiomyocytes contractile events (including systolic and diastolic function) through a process known as excitation-contraction coupling^12^. It is well documented that inappropriate Ca^2+^ homeostasis in ventricular cardiomyocytes are associated with the ventricular tachycardia. Re-opening of L-type Ca^2+^ channels (LTCCs) or other depolarizing currents before normal repolarization completes contributes to the early afterdepolarization (EAD). Gain-of-function mutations on Cav1.2 (calcium channel subtype 1.2) produces Timothy syndrome which characterized by a heart condition similar to LQT syndrome^13^,^14^. Aberrant spontaneous, diastolic Ca^2+^ leakage from the sarcoplasmic reticulum due to point mutation on type 2 ryanodine receptors (RyR2) contributes to formation of delayed after-depolarization (DAD) which leads to heart failure and catecholaminergic polymorphic ventricular tachycardia (CPVT)^15^. In addition to governing contractile events, dysregulation of intracellular Ca^2+^ also undergoes derangements that promote arrhythmogenesis through Ca^2+^ -dependent and coupled electrophysiological effects. Aberrant Ca^2+^ signals can modulate CaMKII activity which in turn regulates the activity of a variety of ion channels and transporters, for examples Nav1.5^16^, RyR2, and SERCA2a^17^,^18^.

In this study, we systematically examined the influence of an array of ion channel modulators on SCO patterns by detecting the intracellular Ca^2+^ dynamics in primary cultured rat ventricular cardiomyocytes using Fluorescence Imaging Plate Reader (FLIPR) in 96-well format. We demonstrate that modification of distinct ion channels differentially affects SCO patterns. In addition, we demonstrate that clinically-used drugs including cisapride, dofetilide, sotalol, and quinidine which cause LQT syndrome all produce characteristic SCO bursts therefore prolong the SCO/burst duration. Our results demonstrate that occurrence of SCO bursts may have a translational value to predict cardiotoxicants causing LQT syndrome.

## Results

### Cultured rat cardiomyocytes displayed spontaneous Ca^2+^ oscillations

**Figure 1:**
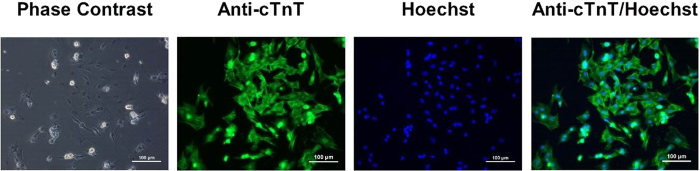
Representative pictures from phase contrast and immunofluorescence staining of neonatal rat ventricular cardiomyocytes after 24 h culture. After 24 h, the cultured cardiomyocytes displayed characteristics of elongated and triangular shape (Phase Contrast). The immunocytochemistry demonstrated that the percentage of cardiomyocytes in our culture system was greater than 95% using a specific cardiomyocytes maker antibody (anti-cTnT) and Hoechst double staining. The experiments were repeated in three independent cultures with similar results.

After 24 h, cultured cardiomyocytes displayed elongated and triangular morphology (Fig. 1). The percentage of cardiomyocytes in our culture system was greater than 95% demonstrated by double staining with anti-cTnT, a specific cardiomyocyte marker, and Hoechst 33342 (Fig. 1). The cultured cardiomyocytes displayed spontaneous beating 24 h after plating which can be observed in a phase-contract microscopy (**data not shown**). It has been well established that the spontaneous beating was correlated with SCO generation^19^ in primary cardiomyocyte cultures. We therefore examined SCOs using rapid throughput machine, FLIPR^®TETRA^. FLIPR^®TETRA^ can simultaneous record the fluorescence intensity from the center area of each well in 96- or 384- well formats therefore representing an overall Ca^2+^ signals from a population of cells. At a plating density of 1.5 × 10^5^ cells/well and after cultured for 60 h, cardiomyocytes displayed rhythmic SCOs, with minimum variability in the frequency, amplitude as well as full width at half maximum (FWHM) (Supplemental Fig. 1). The frequency, amplitude and FWHM were stable during the whole recording time which was 800 s (Supplemental Fig. 2). It should be noted that different batches of ventricular cardiomyocyte cultures showed certain degree of variability in SCO frequency, amplitude as well as FWHM (Supplemental Fig. 2). To diminish the variability, the response on SCO of each compound was normalized to the individual basal parameters (frequency, amplitude and FWHM) from an initial 100 s recording, respectively.

### Epinephrine and acetylcholine affected SCO frequency in primary cultured rat cardiomyocytes

To test whether the source of the SCOs is from extracellular Ca^2+^ influx or intracellular Ca^2+^ store release in our culture system, the cells were bathed in normal extracellular Ca^2+^ (2.3 mM), decreased extracellular Ca^2+^ (1.15 mM) as well as 0 Ca^2+^ [no ethylene glycol-bis(β-aminoethyl ether)-N,N,N’,N’-tetraacetic acid (EGTA)] and SCOs were recorded. Reducing extracellular Ca^2+^ concentration from 2.3 mM to 1.15 mM significantly decreased SCO mean amplitude with slightly enhanced SCO frequency without changing SCO FWHM. Further decreasing the extracellular cellular Ca^2+^ from 1.15 mM to 0 mM (no EGTA) produced an additional increase in SCO frequency and an additional decrease in SCO amplitude without effect on SCO duration (Supplemental Fig. 3). These data suggested that extracellular Ca^2+^ was required for the generation of SCOs in primary cultured ventricular cardiomyocytes.

**Figure 2:**
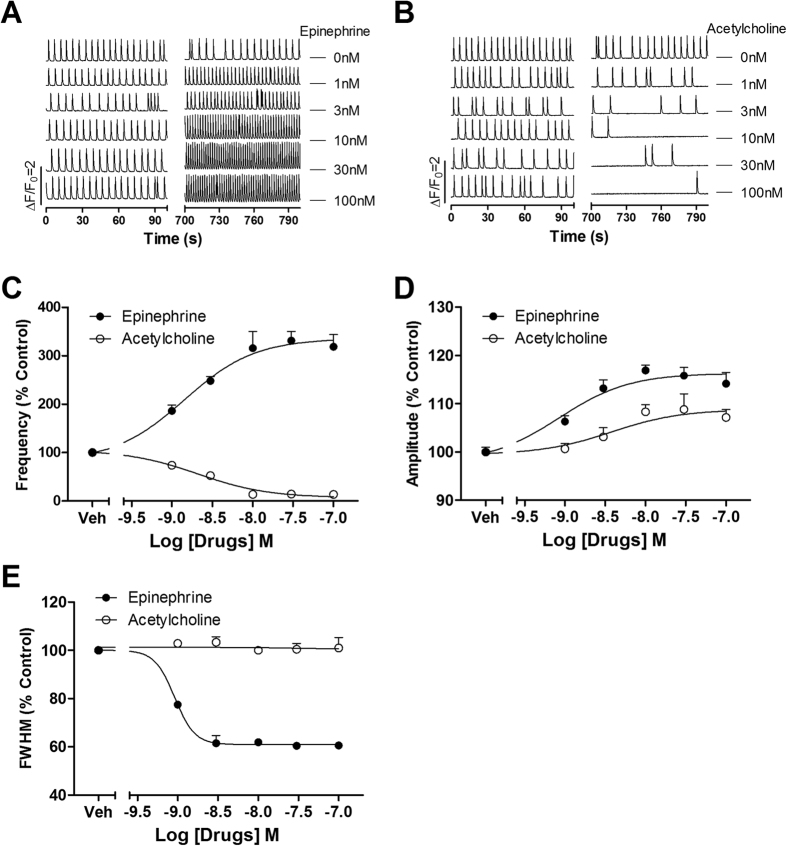
Epinephrine and acetylcholine affected SCO in rat cardiomyocyte cultures. (**A**) Representative traces of epinephrine-induced alterations in Ca^2+^ dynamics. (**B**) Representative traces of acetylcholine-induced alterations in Ca^2+^ dynamics. (**C**) Epinephrine concentration-dependently increased SCO frequency with an EC_50_ value of 1.43 nM (0.75–2.75 nM, 95% CI). Acetylcholine concentration-dependently decreased SCO frequency with an IC_50_ value of 2.10 nM (1.13–3.89 nM, 95% CI). (**D**) Both epinephrine and acetylcholine stimulated SCO amplitude. The EC_50_ values for epinephrine and acetylcholine stimulating SCO amplitude was 0.86 nM (0.33–2.20 nM, 95% CI) and 3.77 nM (0.73–19.4 nM, 95% CI), respectively. (**E**) Epinephrine concentration-dependently decreased the SCO duration with an IC_50_ value of 0.92 nM (0.79–1.06 nM, 95% CI). Acetylcholine was without effect on the SCO duration. The experiments were repeated in three independent cultures each with four replicates.

Acetylcholine and epinephrine can modulate heart beating through activation of β-adrenergic receptors and muscarinic acetylcholine receptors (subtype 2 in cardiomyocytes), respectively^20^. We therefore examined whether these two chronotropes can affect SCOs in our culture system. Figure 2A depicted that epinephrine, a positive chronotrope, concentration-dependently increased both SCO frequency and amplitude. The EC_50_ values for epinephrine stimulating SCO frequency and amplitude were 1.43 nM [0.75–2.75 nM, 95% Confidence Intervals (95% CI)] and 0.86 nM (0.33–2.20 nM, 95% CI), respectively (Fig. 2C,D). Epinephrine also concentration-dependently decreased the SCO duration with an IC_50_ value of 0.92 nM (0.79–1.06 nM, 95% CI) (Fig. 2E). In contrast, acetylcholine, a negative chronotrope, concentration-dependently decreased the SCO frequency with an IC_50_ value of 2.10 nM (1.13–3.89 nM, 95% CI). Acetylcholine also moderately increased the SCO amplitude with EC_50_ value of 3.77 nM (0.73–19.4 nM, 95% CI). Acetylcholine was without effect on the SCO duration (Fig. 2B–E).

### Modification of L-type calcium channel affected SCO pattern in primary cultured rat cardiomyocytes

**Figure 3:**
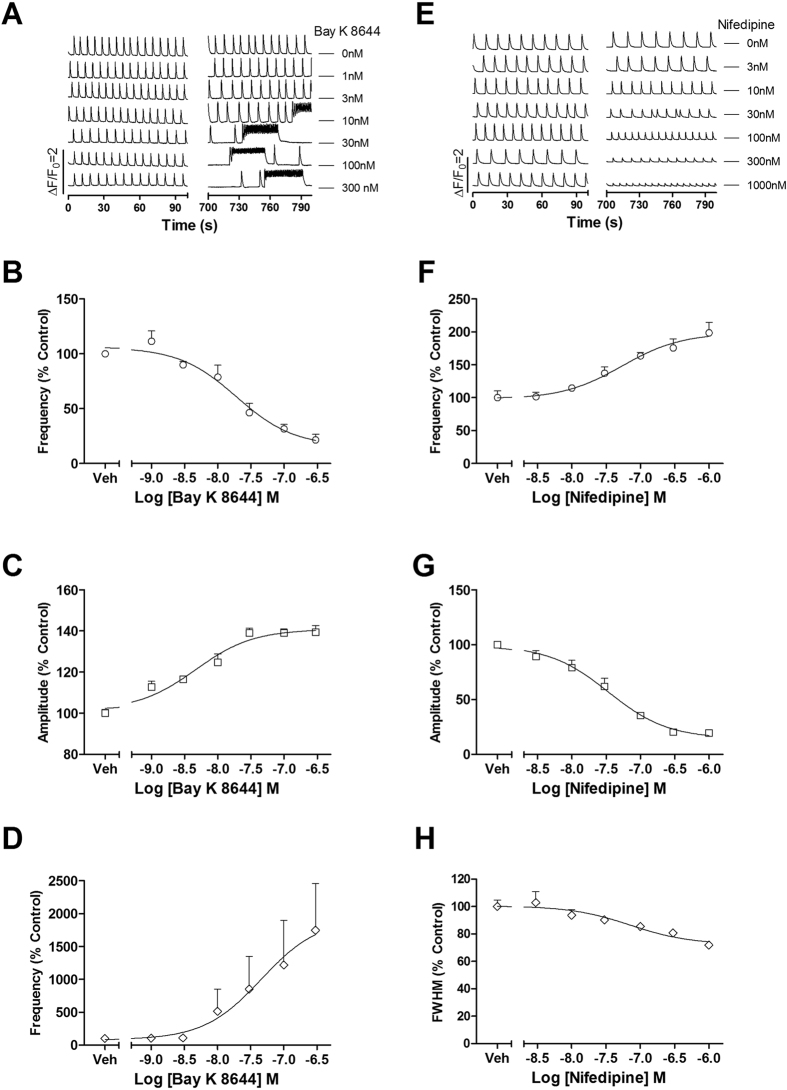
Voltage-gated calcium channel modulators affected SCO pattern in rat cardiomyocyte cultures. Representative traces of Bay K 8644-induced alterations in Ca^2+^ dynamics. Bay K 8644 produced irregularity on the SCO pattern with the occurrence of SCO bursts (**A**). Concentration-response relationship curves of Bay K 8644-induced decrease in frequency (**B**) and increase in amplitude (**C**) as well as prolongation in SCO/burst duration (**D**). Representative traces of nifedipine-induced alterations in Ca^2+^ dynamics (**E**). Concentration-response relationship curves of nifedipine-induced increase in frequency (**F**), decrease in amplitude (**G**) as well as shortening in SCO/burst duration (**H**). The experiments were repeated in three independent cultures each with four replicates.

LTCCs are directly pertinent to the intracellular Ca^2+^ dynamics and are also responsible for the repolarization of the action potentials (APs)^21^. Activation of LTCC by Bay K 8644 produced irregularity on the SCO pattern with the occurrence of SCO bursts accompanied with increased amplitude and longer FWHM (Fig. 3). The EC_50_ values for Bay K 8644 stimulating SCO amplitude and mean SCO/bursts duration were 4.63 nM (2.44–8.78 nM, 95% CI) and 45.7 nM (3.50–599 nM, 95% CI), respectively. Nifedipine, an LTCC inhibitor, concentration-dependently suppressed the SCO amplitude (IC_50_ = 34.1 nM, 18.6–62.6 nM, 95% CI) while slightly increased the SCO frequency (EC_50_ = 53.4 nM, 19.3–149 nM, 95% CI) without significant effect on the SCO duration (Fig. 3).

### Modulation of ryanodine receptors affected SCO pattern in primary cultured rat cardiomyocytes

**Figure 4:**
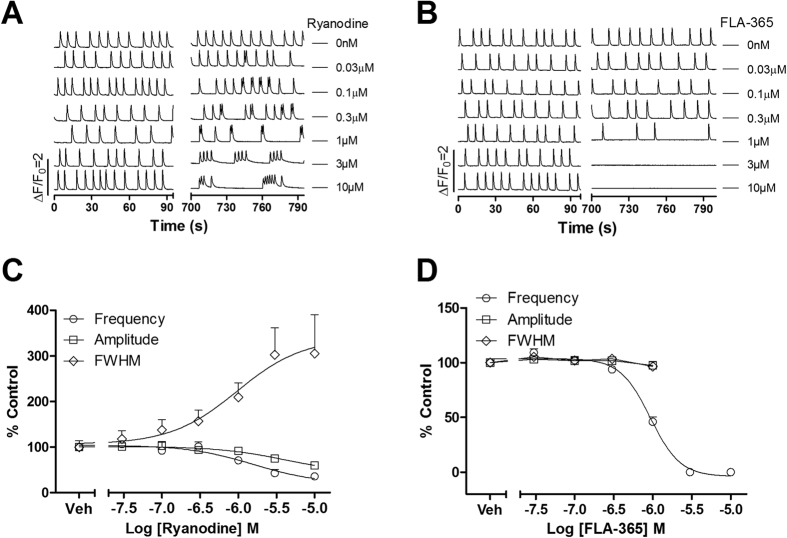
Modulation of ryanodine receptors affected SCO pattern in primary cultured rat cardiomyocytes. (**A**) Representative traces of ryanodine-induced alterations in Ca^2+^ dynamics in cardiomyocytes as a function of time. (**B**) Representative traces of FLA-365-induced alterations in Ca^2+^ dynamics in cardiomyocytes as a function of time. (**C**) Concentration-response relationship curves of ryanodine reduced SCO frequency and amplitude and prolonged FWHM. (**D**) Concentration-response relationship of FLA-365-induced SCO frequency decrease. The experiments were repeated in three independent cultures each with three replicates.

The ryanodine receptor (RyR) is one of the store operated Ca^2+^ channel located in the endoplasmic/sarcoplasmic reticulum and is responsible for Ca^2+^ -induced Ca^2+^ release (CICR). RyR2 is primarily expressed in cardiac tissues. Upon activation following LTCC mediated Ca^2+^ influx, RyR2 releases Ca^2+^ from the sarcoplasmic reticulum into the cytosol, enabling cardiac muscle contraction^22^. FLA-365, a RyR inhibitor^23^, concentration-dependently suppressed the SCO frequency in primary cultured cardiomyocytes (IC_50_ = 0.93 μM, 0.81–1.06 μM, 95% CI) without affecting the SCO duration. Higher concentration of FLA-365 ( ≥3 μM) eliminated SCOs (Fig. 4). Low concentration of ryanodine, which activated RyRs, also modulated the SCO patterns. Application of ryanodine produced SCO bursts (Fig. 4) with increased SCO/burst FWHM.

### Modulation of voltage-gated sodium channel influenced SCO pattern in primary cultured rat cardiomyocytes

**Figure 5:**
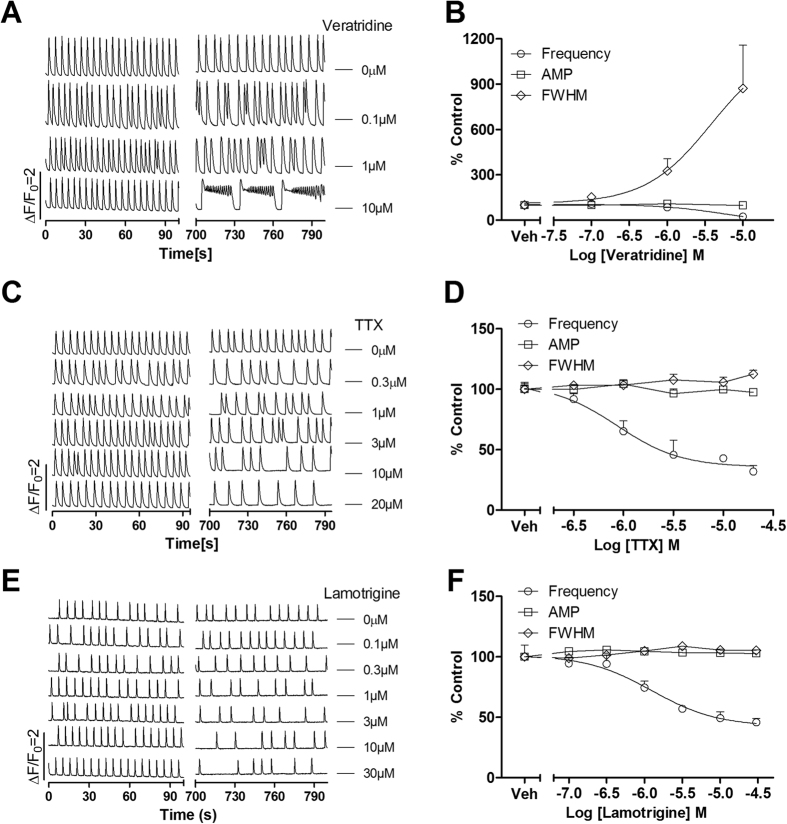
Modification of voltage-gated sodium channel influenced SCO pattern in rat cardiomyocyte cultures. (**A**) Representative traces of veratridine-induced alterations in Ca^2+^ dynamics in cardiomyocytes as a function of time. (**B**) Concentration-response relationship curves of veratridine-induced decrease in SCO frequency and increase in SCO/burst duration. Veratridine had no effect on SCO amplitude. (**C**) Representative traces of TTX-induced alterations in Ca^2+^ dynamics as a function of time. (**D**) Concentration-response relationship curves of TTX-induced reduction in SCO frequency. (**E**) Representative traces of lamotrigine-induced alterations in Ca^2+^ dynamics as a function of time. (**F**) Concentration-response relationship curves of lamotrigine-induced reduction in SCO frequency. The experiments were repeated in three independent cultures each with five replicates.

VGSCs are responsible for the AP generation which is required for the rhythmic cardiomyocyte contraction for cardiomyocytes^21^. We therefore examined the influence of both VGSC agonist, veratridine and the blocker, tetrodotoxin (TTX) on the SCOs. Veratridine at 1 μM significantly increased the SCO FWHM. Veratridine at 10 μM produced SCO bursts which lasted for at least 20 s (Fig. 5A & C). Higher concentration ( ≥ 20 μM) of veratridine disrupted the SCOs and produced a sustained intracellular Ca^2+^ overloading (**Data not shown**). TTX concentration-dependently suppressed the SCO frequency with IC_50_ value of 0.86 μM (0.36–2.05 μM, 95% CI) without changing SCO amplitude and duration (Fig. 5B & D). Application of lamotrigine produced similar response with that of TTX. Lamotrigine suppressed SCO frequency with IC_50_ value of 1.2 μM (0.19–4.74 μM, 95% CI) without affecting SCO amplitude and duration (Fig. 5D).

### Inhibition of voltage-gated potassium channels affected SCOs in primary cultured rat cardiomyocytes

**Figure 6:**
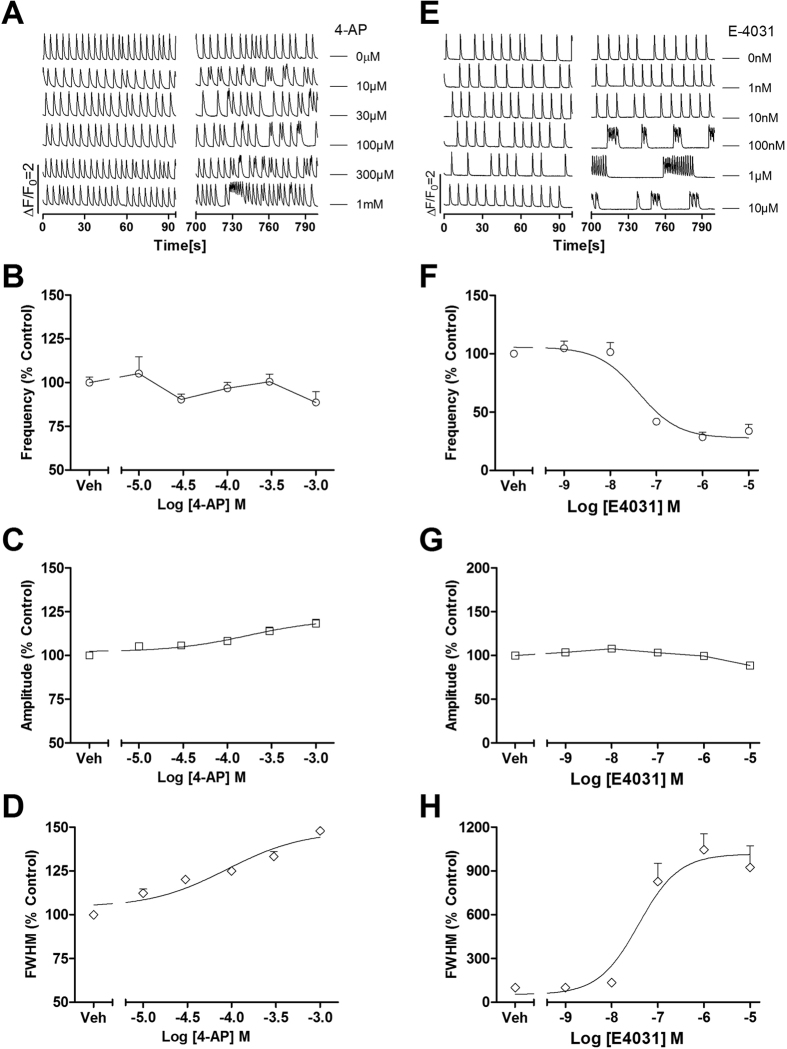
4-AP and E-4031 affected SCOs in rat cardiomyocyte cultures. (**A**) Representative traces of 4-AP-induced alterations in Ca^2+^ dynamics as a function of time. 4-AP produced irregularity on SCO pattern with occurrence of SCO bursts. (**B**) 4-AP was without effect on SCO frequency. (**C**) Concentration-response relationship curve for 4-AP altered SCO amplitude. (**D**) Concentration-response relationship curve for 4-AP altered SCO/burst duration (FWHM). (**E**) Representative traces of E-4031-induced alterations in Ca^2+^ dynamics as a function of time. (**F**) Concentration-response relationship curve of 4-AP suppressing SCO frequency. (**G**) E-4031 did not affect SCO amplitude. H, Concentration-response relationship curve for 4-AP increased SCO/burst duration (FWHM). The experiments were repeated in three independent cultures each with three replicates.

Kvs are responsible for the repolarization of the AP^21^. 4-aminopyridine (4-AP) is a universal Kv channel blocker^24^. As shown in Fig. 6A, 4-AP elicited irregular SCO pattern with the occurrence of SCO bursts. 4-AP increased the mean SCO/ burst duration and slightly increased SCO amplitude with EC_50_ values of 170 μM (48.1–598 μM, 95%CI) and 88.7 μM (28.6–2745 μM, 95%CI), respectively. 4-AP had no effect on SCO frequency (Fig. 6B–D). The Kv11.1 (hERG) channel (encoded by human *Ether-à-go-go* Related Gene) mediates the repolarizing I_Kr_ current in the cardiac APs. Loss of hERG channel function by pharmacological inhibition or genetic mutation has been shown to delay the AP repolarization and widen the ventricular AP, which is the underlying physiological abnormality in LQT syndrome^25^. N-[4-[1-[2-(6-Methylpyridin-2-yl)ethyl]piperidine-4-carbonyl]phenyl] (E-4031), a hERG channel blocker, at concentrations ≥100 nM, produced SCO bursts with long quiescent time between two SCO bursts (Fig. 6E). The EC_50_ values for E-4031 increasing SCO burst duration and decreasing SCO frequency were 38.4 nM (12.2–121 nM, 95% CI) and 40.6 nM (17.6–93.5 nM, 95% CI), respectively (Fig. 6F & H). E-4031 was without effect on SCO amplitude (Fig. 6G).

### Clinically-used drugs causing LQT syndrome produced characteristic bursts of SCO in primary cultured rat cardiomyocytes

**Figure 7:**
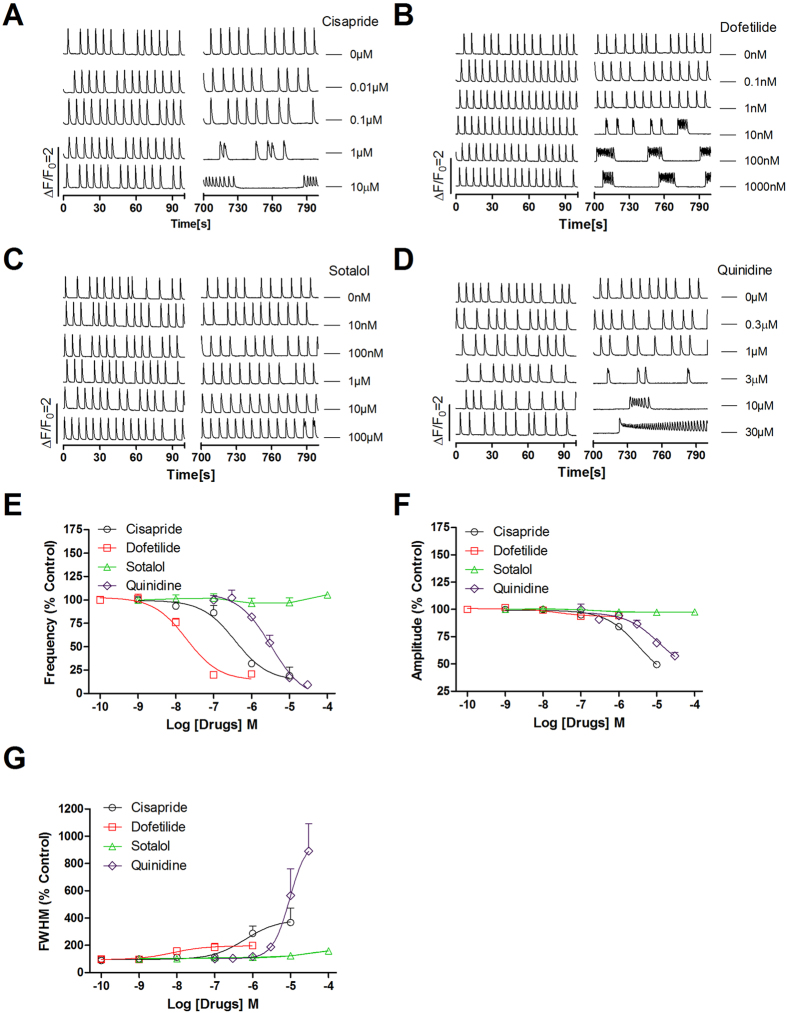
Cisapride, dofetilide, sotalol and quinidine affected SCOs in primary cultured rat cardiomyocytes. Representative traces of cisapride (**A**), dofetilide (**B**), sotalol (**C**) and quinidine (**D**) induced alterations in Ca^2+^ dynamics as a function of time. Concentration-response relationship curves of cisapride, dofetilide, sotalol and quinidine responses in SCO frequency (**E**), amplitude (**F**) and duration (G). This experiment was repeated in three independent cultures each with three replicates.

**Figure 8:**
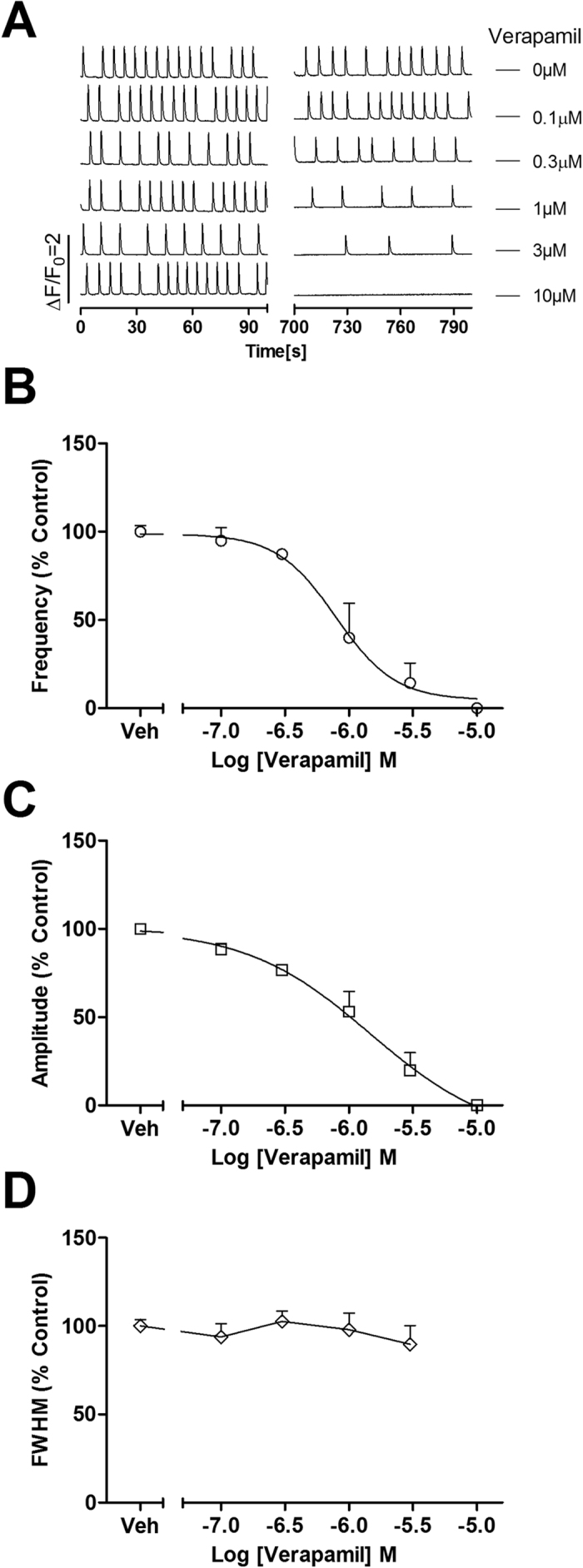
Verapamil affected SCOs in primary cultured rat cardiomyocytes. (**A**) Representative traces of verapamil-induced alterations in Ca^2+^ dynamics as a function of time. Concentration-response relationship curves of verapamil responses in SCO frequency (**B**), amplitude (**C**) and duration (**D**). This experiment was repeated in three independent cultures each with three replicates.

The clinically-used drugs that were withdrawn from the market due to adverse cardiac effect were primarily attributed to their abilities to induce LQT syndrome or TdP^26^. We therefore tested whether those clinically-used drugs which have been reported to cause LQT syndrome including cisapride, dofetilide, sotalol and quinidine could modulate SCO patterns. As shown in Fig. 7, dofetilide, cisapride and quinidine all produced SCO bursts with long quiescent time between two SCO bursts with higher concentration having greater response. The response of sotalol on inducing SCO bursts was somewhat smaller and the SCO bursts only occurred at 100 μM. The EC_50_ values for dofetilide, cisapride, quinidine and sotalol increasing SCO/SCO burst duration were 7.69 nM (2.05–28.9 nM, 95% CI), 0.63 μM (0.10–3.90 μM, 95% CI), 9.38 μM (3.83–22.9 μM, 95% CI), and 27.5 μM (4.26–177.8 μM, 95% CI), respectively (Fig. 8G). Dofetilide, cisapride, and quinidine also concentration-dependently suppressed SCO frequency with IC_50_ values of 19.0 nM (10.4–34.9 nM, 95% CI), 0.36 μM (0.15–0.87 μM, 95% CI) and 3.24 μM (1.73–6.07 μM, 95% CI), respectively. However, sotalol was without effect on the SCO frequency. Cisapride and quinidine also moderately suppressed the SCO amplitude with IC_50_ values of 3.34 μM (2.36–4.72 μM, 95% CI) and 11.3 μM (3.32–38.2 μM, 95% CI), respectively. Verapamil, a dual blocker of hERG channel and LTCC, suppressed the SCO frequency and amplitude with IC_50_ values of 0.79 μM (0.39–1.60 μM, 95% CI) and 1.47 μM (0.50–4.27 μM, 95% CI), respectively. Verapamil was without effect on the SCO duration (Fig. 8).

## Discussion

In this study, using rapid throughput imaging system, we systematically investigated the influence of an array of ion channel modifiers on intracellular Ca^2+^ ([Ca^2+^]_i_) dynamics in primary cultured rat ventricular cardiomyocytes. We demonstrate that altering the activity of Ca^2+^ channels which contribute to [Ca^2+^]_i_ dynamics or ion channels which are involved in AP generation both affect SCO patterns. The potencies of tested reference compounds are generally consistent with their affinities on their respective targets (Table 1) suggesting target-specific Ca^2+^ response in cultured rat ventricular cardiomyocytes. The [Ca^2+^]_i_ not only governs contractile events (including systolic and diastolic function), but also undergoes derangements that promote arrhythmogenesis through Ca^2+^ -dependent and coupled electrophysiological effects^27^. Our data therefore imply that modification of SCO in primary cultured ventricular cardiomyocytes detected by FLIPR^®TETRA^ system may serve as a rapid throughput method capable of identifying potential cardiotoxicants from environment or early stage of drug development.

We demonstrate that acetylcholine dramatically decreases the SCO frequency consisting with previous study where acetylcholine decreases cardiomyocytes contraction resulting from activation of subtype 2 muscarinic acetylcholine receptors^28^. Epinephine, a β-adrenergic receptor agonist dramatically increases SCO frequency which is also consistent with clinically observed heart rate increase^29^. Cardiomyocytes contraction is tightly coupled to SCO which is controlled by APs^12^. The APs, SCOs and contractions of ventricular cardiomyocytes occur simultaneously^19^. Therefore, perturbation of SCO frequency may predict cardiotoxicants which are capable of producing tachycardia or bradycardia.

An important finding is that compounds which contribute to [Ca^2+^]_i_ dynamics or shape APs affect SCO pattern differently. Activation of LTCC by Bay K 8644^30^ induces bursts of SCOs and slightly increases SCO/burst amplitude whereas suppression of LTCC concentration-dependently decreases SCO amplitude and duration with concomitant increase of SCO frequency, a phenomenon observed in the clinic^31^. Activation of RyR by low concentrations of ryanodine ( ≤10 μM)^32^ also produces bursts of SCOs. However, in contrast to increased amplitude by Bay K 8644, ryanodine slightly decreases the SCO/burst amplitude. Suppression of RyR by FLA-365^23^ decreases SCO frequency without affecting SCO amplitude and duration. Higher concentrations ( ≥3 μM) of FLA-365 completely abolish SCOs. Activation of VGSCs which is responsible for AP generation also induces busts of SCOs without changing SCO amplitude. TTX and lamotrigine both decrease SCO frequency but not the amplitude and duration. It should be noted that TTX is a VGSC channel blocker while lamotrigine is a use-dependent VGSC inhibitor. Lamotrigine binds to IVS6 segment of sodium channels^33^ which is topologically distinct from the TTX binding sites^34^. These data suggest that compounds suppressing VGSCs can produce similar SCO profile regardless of their modes of action. Suppression of Kv channels also produces irregularity with prolonged SCO duration and occurrence of SCO bursts. Altogether, these data demonstrate that distinct targets engagements produce different SCO patterns therefore mapping the SCO profiles produced by cardiotoxicants may be capable of predicting their molecular target(s).

Another important finding is that E-4031, a specific Kv11.1 (hERG) blocker, produces SCO bursts and decreases the SCO/burst frequency. The EC_50_ and IC_50_ values of E-4031 on SCO/burst duration and frequency are 38.3 nM and 40.6 nM, respectively, which were consistent with its affinity on hERG channels^35^. Blockade of hERG channel is proposed to be the major cause of LQT syndrome of many withdrawn clinically-used drugs^36^. We demonstrate that four clinically-used drugs including cisapride, dofetilide, sotalol and quinidine that were withdrawn due to their ability to produce LQT also induce SCO bursts in ventricular cardiomyocyte cultures. It should be mentioned that cisapride, dofetilide, and sotalol directly inhibit hERG channel activity whereas quinidine decreases the surface expression of hERG channels by suppressing hERG channel translocation to cell membrane surface^37^. These data imply that regardless of mode of action, functional suppression of hERG channel activity by cardiotoxicants produces similar characteristic SCO prolongation/occurrence of SCO bursts. The potencies for dofetilide and sotalol increasing SCO/burst duration are comparable with their affinities on hERG channels^38^,^39^. However, the EC_50_ values for cisapride and quinidine are significantly deviated from their affinities to hERG channels^40^,^41^. Cisapride has been demonstrated to affect many other channels such as Kv4.1^42^ and receptors such as 5-HT4 receptors^43^. In addition to inhibiting hERG, quinidine inhibits both Na^+^ and Ca^2+^ currents^44^,^45^. The integrated response from many ion channels interaction may account for potency difference between hERG blockade and increased SCO duration. Verapamil is an anti-hypertension drug used in the clinic. Although verapamil is a potent hERG channel blocker, no evidence exists that verapamil can produce LQT syndrome^21^. We demonstrate that verapamil has no effect on inducing SCO bursts although it decreases SCO frequency. The later response is likely derived from suppression of LTCC^46^. Therefore, our data demonstrate that not all the hERG channel blockers are capable of inducing SCO busts in primary cultured rat ventricular cardiomyocytes and only those compounds producing LQT syndrome can stimulate SCO.

In addition to clinically-used drugs, we also demonstrate that sodium channel agonist, veratridine and LTCC activator, Bay K 8644 as well as the universal potassium channel blocker, 4-AP produce SCO bursts. It has been demonstrated that veratridine prolongs AP duration in rabbit ventricular myocytes^47^. Although inactive on hERG channels, alfuzosin produces LQT syndrome in the clinic due to its ability to increase the peak current of Nav1.5^48^. Genetic mutations in ion channels also lead to LQT syndrome. Gain of function of VGSCs mutations leads to LQT syndrome and Brugada syndromes^6^,^7^. Loss-of-function of Kv channel conductance results in AP prolongation leading to LQT syndrome^8^. Gain-of-function mutations on Cav1.2 produce Timothy syndrome which is characterized by a heart condition similar to LQT syndrome^14^. Therefore, these data together demonstrate that induced SCO bursts may predict the compounds with ability to induce LQT syndrome. A large set of LQT positive and negative reference compounds are required to be investigated to fully establish the relationship between prolonged SCO/burst duration and LQT and how accuracy of this model to predict the compounds with ability to induce LQT syndrome.

A variety of compounds-induced SCO patterns have been investigated in induced pluripotent stem cell (iPSC)-derived cardiomyocytes (iPSC-CMs). The hERG channel inhibitors cisapride, terfenadine, astemizole, and pimozide, all characteristically delay SCO recovery^49^. Sodium channel blockers lidocaine and tetrodotoxin slow beating frequency and various other irregularities^49^. Drug with positive chronotropic effect stimulates the SCO frequency^49^. In contract to our observation in cultured rat cardiomyocytes where acetylcholine decreases SCO frequency, no response was observed when iPSC-CMs were challenged with acetylcholine suggesting that iPSC-CMs lacks M2 receptor signaling pathways^49^. Despite the similarity in the expression of specific cardiac biomarkers, electrophysiology, and pharmacology^50^, when comparing hiPSC-CMs to human cardiac tissue, the immature electrophysiological properties and Ca^2+^ handling of iPSC-CM were reported^51^. In addition, recent studies have pointed out the limitations of using iPSC-CMs on drugs screening and toxicity test. The cost of iPSC-CMs is currently still high which is an important consideration when using iPSC-CMs for cardiotoxicity screening^21^. Second, the purity of iPSC-CMs is typically less than 75% and the functional status of iPSC-CMs only exists for a short period^52^. Furthermore, iPSC-CM is more sensitive to DMSO exposure with 0.1% DMSO affecting contractile amplitude and beating rates which may also affects the results explanation^53^.

In summary, we demonstrate that modification of distinct targets differentially affects the SCO pattern in primary rat cardiomyocyte cultures. Although a large set of LQT positive and negative compounds were required to be investigated to fully establish the relationship between LQT and SCO bursts, we demonstrate that clinically-used drugs which produce LQT all produce SCO bursts while negative drugs do not. Therefore, this method may serve as a primary screening for detecting the cardiotoxicants in the early stage of drug development. In addition, mapping the profiles induced by cardiotoxicants may predict their molecular target(s). More importantly, this method may have translational value for detecting the compounds which have the potential to cause LQT syndrome.

## Material and Methods

### Materials

Bay K 8644, veratridine, terfenadine, cisapride, anti-cTnT (1:400) antibody, E-4031 and verapamil were purchased from Abcam (Cambridge, MA, USA). Quinidine was from Tocris Bioscience (Bristol, UK). Nifedipine, 4-aminopyridine, fetal bovine serum (FBS), cytosine β-D-arabinofuranoside (ARA-C), poly-D-lysine and epinephrine, FLA-365, ryanodine, lamotrigine, carbamazepine, phenytoin and all inorganic salts were purchased from Sigma-Aldrich (St. Louis, MO, USA). Bovine serum albumin, DMEM/F12, Triton™ X-100, Alexa Fluor^®^ 488-conjugated goat anti-rabbit were from ThermoFisher Scientific (Waltham, MA). The Ca^2+^ dye Fluo-8/AM was from TEFLab (Austin, TX, USA).

### Primary Cultures of Neonatal Rat Cardiomyocytes

Animal experiments were conducted in accordance with the guidelines of Animal Use and Care of the National Institutes of Health approved by the Institutional Animal Care and Use Committee of China Pharmaceutical University. Sprague-Dawley rat were supplied by Qing-Long-Shan animal center (Nanjing, Jiangsu, China). Cardiomyocytes were dissociated from ventricles dissected from Sprague-Dawley rat pups at postnatal day 3 and maintained in cold dissection buffer (in mM: NaCl 137, KCl 5.36, MgSO_4_ 0.81, dextrose 5.55, KH_2_PO_4_ 0.44, Na_2_HPO_4_ 0.34, HEPES 20, pH 7.4). After centrifugation at 200 g for 5 min, the cells were resuspended in the culture medium (DMEM/F12 supplemented with 20% FBS, 10 μM ARA-C, 100 units/mL penicillin and 0.1 mg/mL streptomycin). Dissociated myocardial cells were seeded in 60 mm petri-dishes for 45 min to remove the fibroblast cells. The unattached cells (enriched cardiomyocytes) were then plated onto poly-D-lysine (20 μg/mL) coated clear-bottom, black wall, 96-well plate (Corning, NY 14831, USA) at a density of 1.5 × 10^5^ cells/well in the culture medium. The medium was changed after 24 h culture by replacing half volume of the medium by fresh culture medium. The cells were maintained at 37 °C with 5% CO_2_ and 95% humidity until used.

### Measurement of Intracellular Calcium Dynamics

Cardiomyocyte cultures at 2 days *in vitro* (DIV) were used to investigate the basal characteristics of intracellular calcium dynamics and how ion channel ligands or arrhythmogenic drugs alter these signals. Briefly, the growth medium was removed and replaced with dye loading buffer (50 μL/well) containing 4 μM Fluo-8/AM, 0.5% bovine serum albumin in pre-warmed Locke’s buffer (in mM: HEPES 8.6, KCl 5.6, NaCl 154, glucose 5.6, MgCl_2_ 1.0, CaCl_2_ 2.3, and glycine 0.1, pH 7.4). After 45 min incubation in dye loading buffer, cells were washed four times by adding and removing 150 μL fresh Locke’s buffer repeatedly. The final volume of each well was adjusted to be 175 μL. After loading, the plate was then transferred to a Fluorescence Imaging Plate Reader (FLIPR^®TETRA^, Molecular Devices, Sunnyvale, CA) chamber which was pre-warmed to 37 °C. Cells were excited at 488 nm, and Ca^2+^ -bound Fluo-8 emitted fluorescence at 515–575 nm range was recorded. The basal fluorescence was recorded for approximately 300 s at a sampling rate of 8 Hz. After recording the basal fluorescence, the drugs (25 μL, 8X) were added to different wells by an automated, programmable pipetting system yielding a final volume of 200 μL/well. The fluorescence was recorded for additional 500 s at a sampling rate of 8 Hz. The fluorescence data were presented as ΔF/F_0_, where F is the fluorescence signal at different time point minus background fluorescence whereas the F_0_ is the basal fluorescence minus the background fluorescence. The background fluorescence was from the sister wells which were not loaded with Fluo-8/AM. To quantify the drug influence on the SCOs, we analyzed SCO frequency, peak amplitude, as well as SCO duration (FWHM). Many drugs induced SCO bursts and in this case, the SCO duration was calculated as SCO burst duration. A burst was defined to be a cluster of SCOs (at least two) where the previous SCO can’t recover to below 20% of the previous SCO amplitude when followed SCO occurred. When drugs induced SCO bursts, a burst was count as an SCO.

### Immunofluorescence

Cardiomyocytes at 2 DIV were fixed with 4% paraformaldehyde for 15 min and then permeabilized with 0.25% Triton™ X-100 for 15 min under gentle shaking. After blocking with 5% goat serum in PBS for 30 min, cells were incubated with anti-cTnT (1:400) antibody overnight at 4 °C. Cells were then incubated with Alexa Fluor^®^ 488-conjugated goat anti-rabbit (1:500) secondary antibody for 2 h at RT. After aspirating secondary antibody, a concentration of 2 μg/mL of Hoechst 33342 was added to stain the nuclei. Images were taken using a Nikon eclipse fluorescence microscope using FITC filter and DAPI filter.

### Data Analysis

Graphing and statistical analysis were performed using GraphPad Prism software (Version 5.0, GraphPad Software Inc., San Diego, CA, USA) and Origin Pro 8 (Origin Lab Corporation, USA). Peaks were fit by Origin Pro 8 (Origin Lab Corporation, USA). The EC_50_ values and the 95% confidence intervals were determined by non-linear regression using GraphPad Prism (Version 5.0, GraphPad Software Inc., San Diego, CA, USA). Statistical significance between different groups was calculated using an ANOVA and, where appropriate, a Dunnett’s multiple comparison test; *p* values below 0.05 were considered to be statistically significant.

## Additional Information

**How to cite this article:** Li, X. *et al*. Modification of distinct ion channels differentially modulates Ca^2+^ dynamics in primary cultured rat ventricular cardiomyocytes. *Sci. Rep.* **7**, 40952; doi: 10.1038/srep40952 (2017).

**Publisher's note:** Springer Nature remains neutral with regard to jurisdictional claims in published maps and institutional affiliations.

## Supplementary Material

Supplementary Dataset

## Figures and Tables

**Table 1:** Comparison of potencies of ion channels modifiers tested on SCO responses and that from their respective targets.

Compounds	Targets	IC_50_/EC_50_ values (95% CI, μM)			Potency (μM)	Ref.
		Frequency	Amplitude	FWHM		
Veratridine	VGSC	5.79 (0.48–69.79)	—	3.83 (0.029–506.8)	4.76	54
TTX		0.86 (0.36–2.05)	—	—	1.52	55
4-AP	Kv	—	170 (48.1–598)	88.7 (28.6–2745)	100	56
E-4031	Kv11.1	0.041 (0.017–0.093)	—	0.038 (0.012–0.121)	0.099	57
Bay K 8644	LTCC	0.019 (0.009–0.040)	0.005 (0.002–0.009)	0.046 (0.035–0.599)	0.032	58
Nifedipine		0.053 (0.019–0.149)	0.034 (0.019–0.063)	0.071 (0.017–0.299)	0.022	59
Ryanodine	RyR2	1.57 (0.37–6.76)	3.60 (1.79–7.23)	0.96 (0.14–6.52)	~1	32
FLA-365		0.93 (0.81–1.06)	—	—	1–1.5	23

“—” means no response.
