# Supplementary material for: Modification of distinct ion channels differentially modulates Ca2+ dynamics in primary cultured rat ventricular cardiomyocytes
Source: Sci Rep. 2017 Jan 19;7:40952. doi: 10.1038/srep40952 (PMC5244425; doi:10.1038/srep40952)
Supplement: Supplementary Dataset [file srep40952-s1.doc]

**Modification of distinct ion channels differentially modulates Ca^2+^ dynamics in primary cultured rat ventricular cardiomyocytes**

Xichun Li^+^, Liping Shen^+^, Fang Zhao, Xiaohan Zou, Yuwei He, Fan Zhang, Chunlei Zhang^*^, Boyang Yu, and Zhengyu Cao^*^


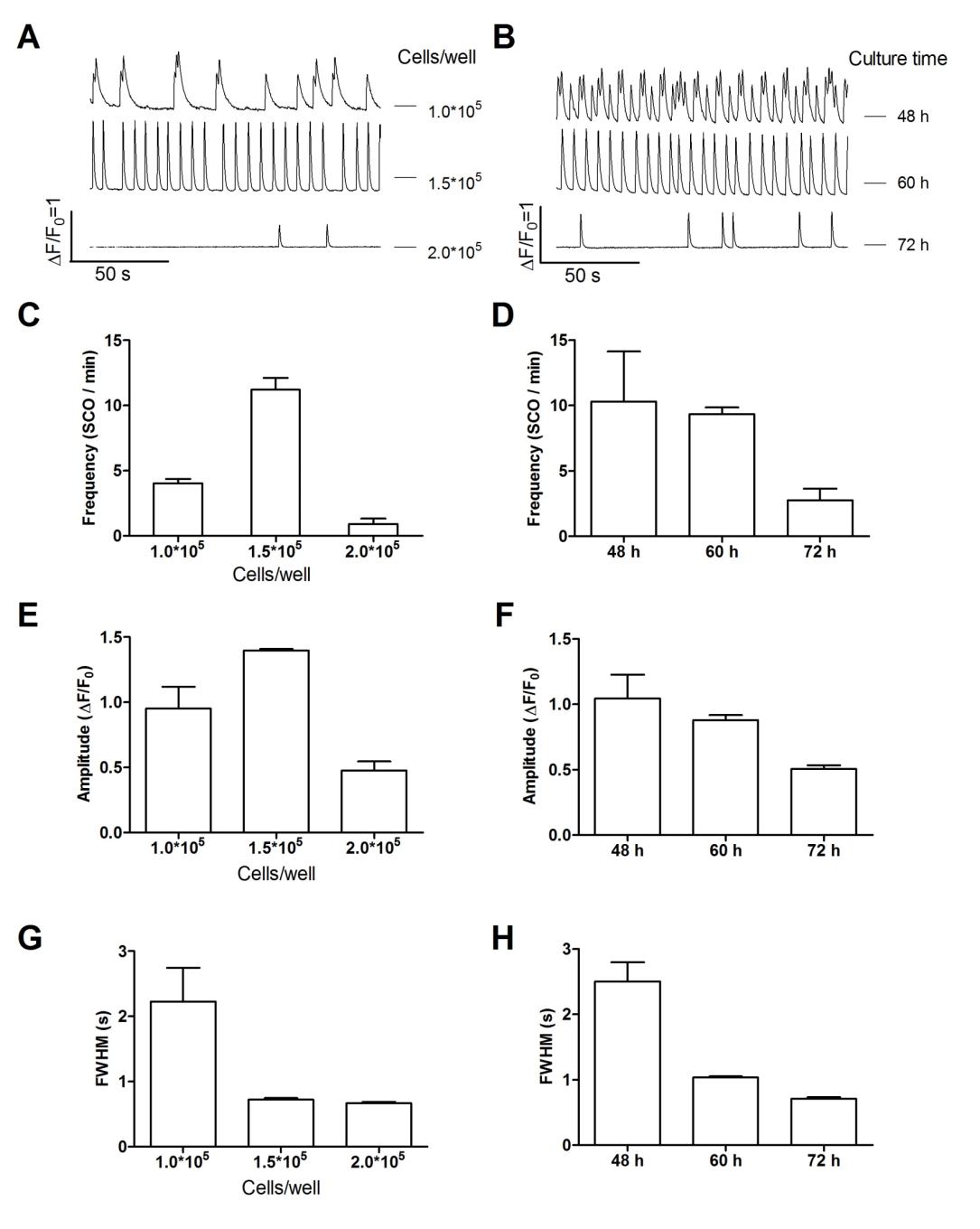


**Supplemental Figure 1 |**Optimization of plating densities (A) and culture times (B) for cardiomyocytes to form rhythmic and synchronous spontaneous Ca^2+^ oscillations (SCOs). Quantification of SCO frequency (C), amplitude (E) and SCO duration (G) of ventricular cardiomyocyte cultured at 60 h in different plating densities. Quantification of SCO frequency (C), amplitude (E) and SCO duration (G) of ventricular cardiomyocyte cultured at different times in an initial plating density of 1.5*10^5^ cells/well in a 96-well format.


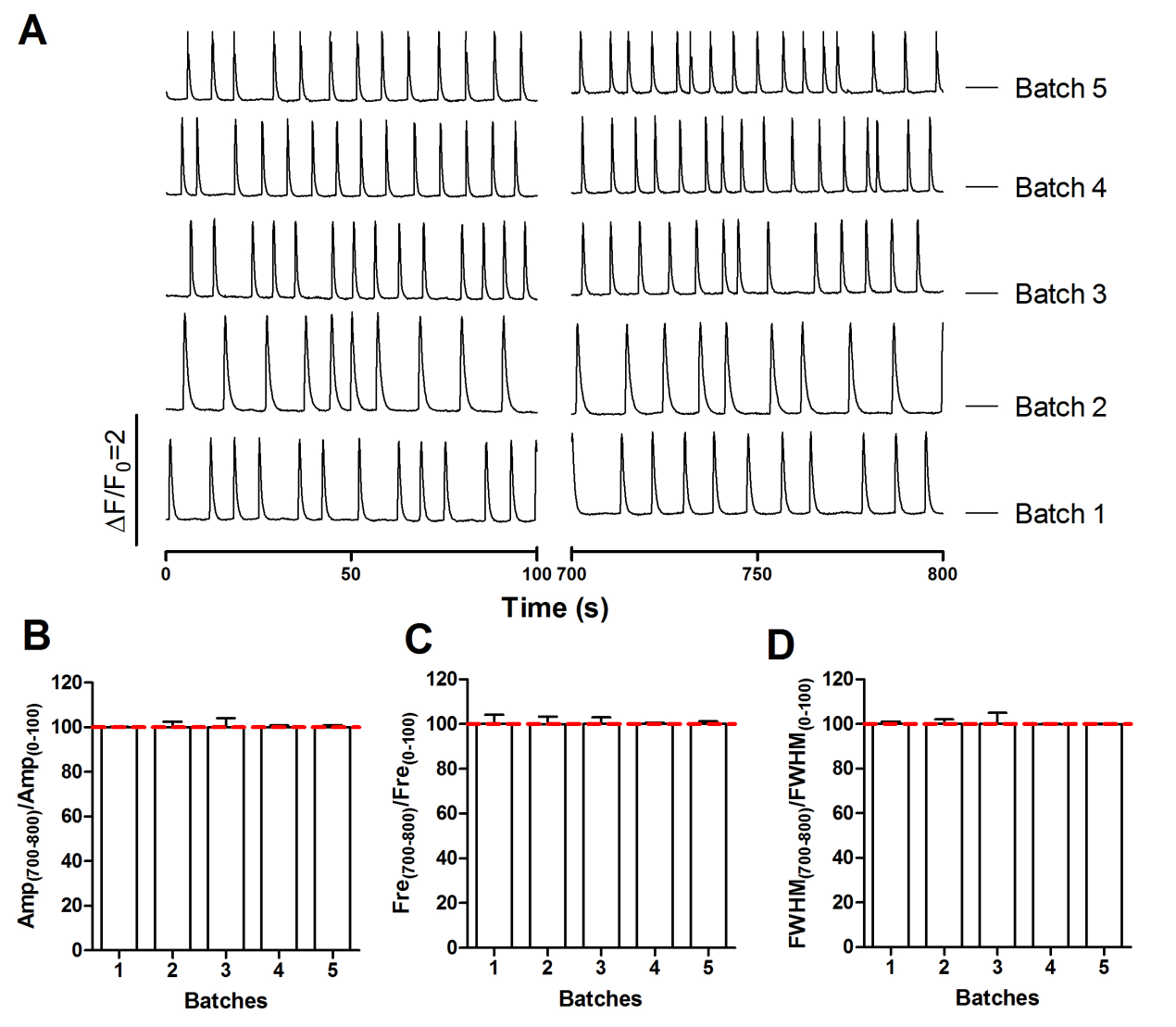


**Supplemental Figure 2 |Batch to batch repeatability and stability of SCO during a recording epoch of 800 s in ventricular cardiomyocyte cultures.** (A) The representative SCOs from five batches of cultures recorded for 800 s. Quantification of the SCO frequency (B) amplitude (C) and full width at half maximum (FWHM) (D) in the initial 0-100 s and 700-800 s. The SCOs are stable during the recording times (800 s).


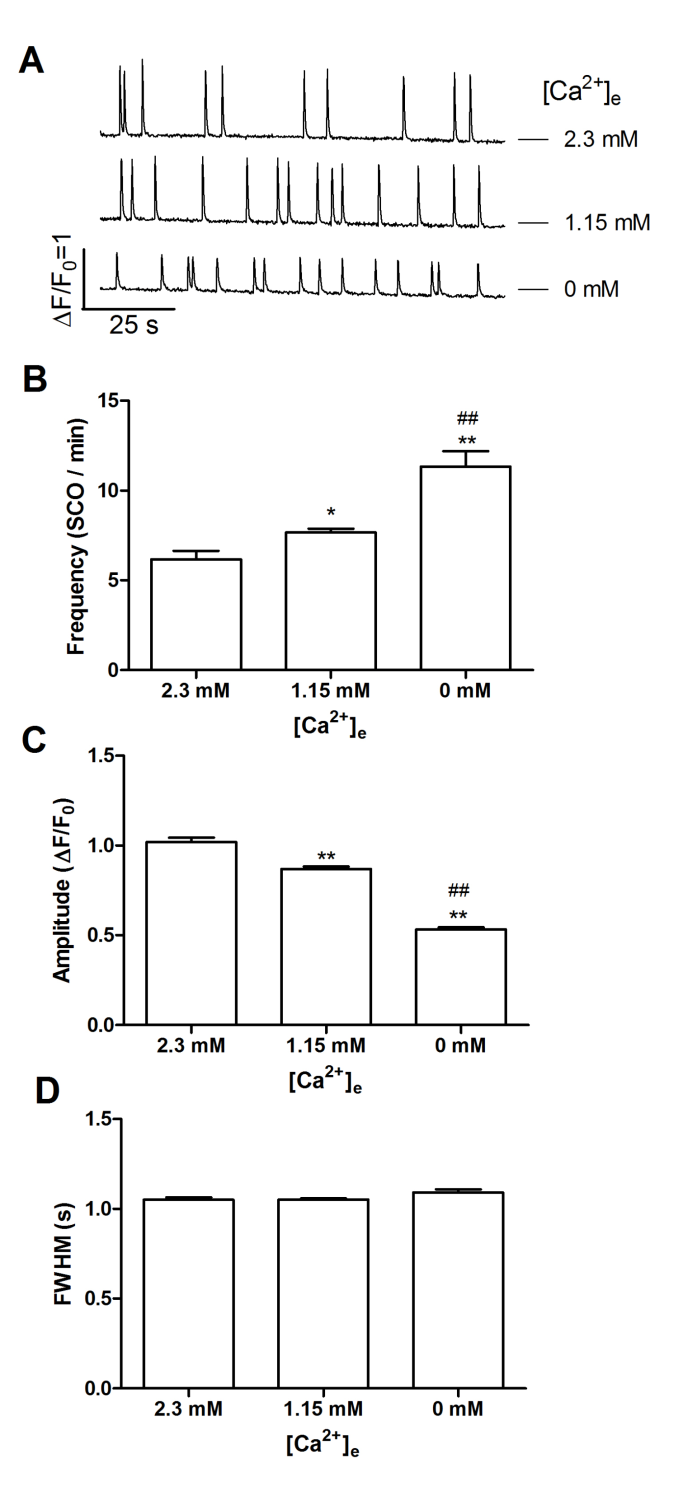


**Supplemental Figure 3** **|**SCOs in rat ventricular cardiomyocyte cultures are dependent on the extracellular Ca^2+^ concentrations. (A) Representative traces of SCOs in normal (2.3 mM), medium (1.15 mM) and low (0 mM, w/o EGTA) extracellular Ca^2+^ concentrations. Quantification of the SCO frequency (B) amplitude (C) and duration (FWHM) (D) in normal (2.3 mM), medium (1.15 mM) and low (0 mM, w/o EGTA) extracellular Ca^2+^ concentrations.
